# A review of the diet, nutrients, and supplementation potential for the outcome augmentation in surgical treatment of peripheral nerve injuries

**DOI:** 10.3389/fsurg.2022.942739

**Published:** 2022-11-09

**Authors:** Sanja Lepić, Milan Lepić, Nikolina Banjanin, Stefan Mandić-Rajčević, Lukas Rasulić

**Affiliations:** ^1^ Institute of Hygiene, Military Medical Academy, Belgrade, Serbia; ^2^ Faculty of Medicine of the Military Medical Academy, University of Defense, Belgrade, Serbia; ^3^ Clinic for Neurosurgery, Military Medical Academy, Belgrade, Serbia; ^4^ Institute of Hygiene and Medical Ecology, Faculty of Medicine, University of Belgrade, Belgrade, Serbia; ^5^ School of Public Health and Health Management and Institute of Social Medicine, Faculty of Medicine, University of Belgrade, Belgrade, Serbia; ^6^ Faculty of Medicine, University of Belgrade, Belgrade, Serbia; ^7^ Department for Peripheral Nerve Surgery, Functional Neurosurgery and Pain Management Surgery, Clinic for Neurosurgery, University Clinical Center of Serbia, Belgrade, Serbia

**Keywords:** nutrition, supplements, peripheral nervous system, injury, surgery, regeneration, functional recovery

## Abstract

**Objective:**

Although the studies have shown the beneficial effects of diet, nutrition, and supplementation as an independent treatment modality, their roles are underestimated in the treatment of peripheral nerve injuries. This is in great part due to the development of efficient nerve repair techniques, combined with physical treatment and stimulation. To achieve the best possible functional recovery diet, nutrition, and supplementation should be implemented within a multidisciplinary approach. The aim of the study is to provide insight into the potentially beneficial effects of diet, nutrients, and supplementation, in the limitation of nerve damage and augmentation of the functional recovery after surgery in a review of human and animal studies.

**Methods:**

The data relating to the diet, nutrients, and supplementation effects on peripheral nerve injuries and their treatment was extracted from the previously published literature.

**Results:**

General balanced diet as well as obesity influence the initial nerve features prior to the injury. In the period following the injury, neuroprotective agents demonstrated beneficial effects prior to surgery, and immediately after the injury, while those potentiating nerve regeneration may be used after the surgical repair to complement the physical treatment and stimulation for improved functional recovery.

**Conclusions:**

Standardized diet, nutrition, and supplementation recommendations and protocols may be of great importance for better nerve regeneration and functional recovery as a part of the multidisciplinary approach to achieve the best possible results in surgically treated patients with peripheral nerve injuries in the future.

## 1. Introduction

The development of peripheral nerve (PN) surgery after traumatic injuries had reached the limits of functional recovery through contemporary nerve repair techniques (1). The majority of studies dealing with surgical treatment are limited to the surgical perspective only, less commonly combined with physical treatment, and rarely with stimulation, while the medical and other treatment is usually reserved for those not being candidates for surgery (2).

The understanding of the Schwann cells response and the brain plasticity in PN injuries, together with modern nerve repair strategies had led to enviable functional recovery (3, 4). However, these results are in large part improved by mandatory physical treatment and stimulation (5). Diet, nutrients, and supplements impact on the other hand is observed as a sole treatment modality, usually in animal models with crush injury (6). Although the results are encouraging and the conclusions are extremely positive, human studies are lacking.

Complementary supplementation and nutrition are one more point where one can augment the outcome of repair; however, there are no guidelines, recommendations, or review studies to give nerve specialists another card to play with (7).

To understand the real-life impact of diet, nutrients, and supplementation on functional recovery in humans with injured nerves, we are not allowed to deprive the surgical treatment. Nevertheless, the two modalities are rarely combined on purpose. The augmentation of the functional recovery after a reconstructive surgical procedure, through the adjusted diet and nutrition, with additional supplementation is a perspective (8).

This review aimed to provide insight into the effects of diet, nutrients, and supplements related to PN preservation and regeneration after traumatic injury, as well as to imply the significance of outcome augmentation, in addition to the surgical repair in patients with PN injuries, through a review of animal models studies.

## 2. Methods

The data relating to the supplementation, nutrition and diet effects from the studies on PN injuries was extracted. Studies published in scientific literature included in the databases PubMed, Google Scholar, Science Direct, and Web of Science were evaluated. No limitations in terms of study design were applied. Both human and animal studies were included. Special attention was taken to the implications of outcome augmentation after surgical treatment.

To identify the nutritional factors impacting the recovery of the PN after injury, we have performed an initial search, to identify any review studies on diet, nutrients, and supplements' roles in PN injuries. This led to the identification of these factors for further literature search.

Articles of interest were found through the searches of PubMed, Science Direct, and Google Scholar databases using the keywords peripheral nerve OR brachial plexus OR peripheral nervous system AND injury OR trauma in combination with the common terms [e.g., “diet,” “nutrient(s),” “supplement”, etc.] and the identified factors keywords (e.g., “Vitamin D,” “alcohol,” etc.).

The analysis of cited references led to the inclusion of even more studies, which were omitted from the search results.

### 2.1. Inclusion criteria

• Nerve injury, including peripheral and cranial nerves,
• Either crush or transection injury, and
• Studies in patients or animal models.

### 2.2. Exclusion criteria

• Review papers and
• Combination with pharmacological agents (drugs).

## 3. Review and discussion

Out of the 42 identified publications, we included 34 relevant animal model experiments. Four reviews were excluded, and one human randomized controlled trial. Also, three animal studies were excluded for the combined use of targeted substances with pharmacological agents (drugs). The details of the studies, with the element in scope, the effect and the proposed mechanism are listed in Table 1.

**Table 1:** Diet, nutrients, and supplements playing a role in peripheral nerve injuries with their effects and proposed mechanisms.

	Author(s), date	Subjects	Injured nerve	No. of cases	Effect	Mechanism observed
Diet						
Balanced diet	Opalach et al. (2010)	Rats	Sciatic nerve	12	Maintains a younger state in peripheral nerves	Age-related oxidative damage defy.
Energy value	Rangaraju et al. (2009)	Rats	Sciatic nerve	N/A	The improvements in nerve architecture with diet restriction.	By sustained expression of protein chaperones and markers of the autophagy–lysosomal pathway.
Obesity	Bekar et al. (2014)	Rats	Sciatic nerve	24	Obesity may affect peripheral nerve structure and regeneration negatively after crush injury. Schwann cells were stained darkly and appeared damaged. Myelinated axons had irregular myelin sheaths: Endoneurium was more pronounced Regenerated axons were smaller	Obesity may reduce the amounts of growth factors (GAP-43) or their effectiveness, which results in a delay in regeneration and recovery.
Alcohol	Ertem et al. (2009)	Rats	Posterior tibial nerve	32	Alcohol intake has negative influences on peripheral nerve regeneration: Severe axonal loss Myelin degeneration Regenerative clusters Endoneural fibrosis	Direct toxic effect of alcohol is suggested, although subsequential malnutrition may play a more important role.
Nutrients						
Carbohydrates	Singer and Mehler (1983)	Rats	Hypoglossal nerve	12	A relatively greater amount of glucose is taken up into the regenerating nucleus at the time of 2-deoxyglucose infusion in fasting patients.	Probably due to increased glucose use during fasting, a deficit of glucose occurs in the regenerating nucleus.
Lipids	Liskiewicz et al. (2016)	Rats	Sciatic nerve	51	Regeneration of sciatic nerves was improved in ketogenic diet preconditioned rats.	Ketogenic diet may influence growth of mature nerve fibers, thus improving regeneration.
	Michael-Titus (2007)	Mice	Facial nerve	12	Dietary administration of docosahexaenoic omega-3 fatty acid expresses neuroprotective and pro-regenerative effects after nerve injury.	Significant effect on the response of neurons and microglia to injury, and appears to promote a pro-regenerative response.
	Gladman et al. (2012)	Mice	Sciatic nerve	22	Polyunsaturated fatty acids mediate neuroprotective and pro-regenerative effects, while also inhibiting neuroinflammation and oxidative stress	The exact mechanism is not known, and it is considered to be a combination of targets, from ion channels to nuclear receptors.
	Silva et al. (2017)	Mice	Sciatic nerve	N/A	Oral administration of combined eicosapentaenoic and docosahexaenoic acids accelerates regeneration, prevents neuropathic pain, and possibly expresses protective properties after peripheral nerve injury.	Modulation of glial cells, which are considered the main producers of tumor necrosis factors, may be involved in these effects.
	Avila-Martin et al. (2015)	Rats	Spared Nerve Injury Model^a^	47	Omega-9 fatty acids reduce noxious hyperreflexia and pain-related anxiety behavior following peripheral nerve injury.	Decreases the COX-2/COX-1 ratio in lipopolysaccharide-activated macrophage cells and OX-42 expression within the ipsilateral lumbar spinal dorsal horn
Alpha-lipoic acid	Demir et al. (2014)	Rats	Sciatic nerve	24	Alpha-lipoic acid has a protective effect through the improvement of regeneration of the injured nerve.	Antiapoptotic and anti-inflammatory effects.
	Haidar et al. (2020)	Rats	Sciatic nerve	126	Implatation of composite nanofiber sheets incorporating alpha-lipoic acid and atrovastatin improved both motor and sensory recovery	This novel formulation uses multiple neuroprotective drugs presented together, but with the different release profiles on nerve regeneration.
	Wang et al. (2021)	Rats	Sciatic nerve	48	Alpha-lipoic acid may be used to treat neuropathic pain caused by peripheral nerve injury	Significantly shortened paw withdrawal threshold and latency, improved morphologic changes in the dorsal root ganglia, reduced the aggregation and proliferation of satellite glial cells, and decreased numbers of P53 + cell
Proteins	Pan et al. (2009)	Rats	Sciatic nerve	122	Oral intake of natto increases regeneration (improvement of motor function and in electrophysiological study), decreases vacuole number, increased angiogenesis and axon counts and improves expression of myelin	Decreases injury-induced fibrin deposition, improves injury-induced disruption of blood-nerve barrier and loss of matrix component such as laminin and fibronectin. Attenuates the production of TNF-alpha and apoptosis.
Leucin	Singer and Mehler (1983)	Rats	Hypoglossal nerve	12	Regenerating neurons leucine uptake in fasted animals is increased.	Either due to the deficit of amino acid produced by fasting, inducing or derepressing enzymes which transport leucine, or amino acid metabolism depression because of the deficit of glucose.
Carnosine	Mirzakhani et al. (2018)	Rats	Sciatic nerve	72	The regenerating effect of carnosine on the muscle mass is likely through: acceleration of functional recovery improvement of histological and ultrastructural alterations	Suppression of lipid peroxidation, provokes antioxidant enzyme activity and amelioration of cytokine production.
Acetyl-L-carnitine	Avsar et al. (2014)	Rats	Sciatic nerve	24	Functional recovery of rats treated with acetyl-L-carnitine significantly improved in walking track analysis, and the latencies of the somatosensory evoked potentials components were significantly decreased.	Acetyl-L-carnitine accelerates sciatic nerve regeneration by reducing apoptosis and lipid peroxidation and promoting myelinization.
	Mohammadi et Amini (2017)	Rats	Sciatic nerve	N/A	Acetyl-L-carnitine loaded in a silicone tube bridging the nerve defect improves functional recovery and quantitative morphometric indices of sciatic nerve.	Faster recovery of the axons led to the statistically significant difference between the muscle weight ratios, and morphometric indices showed that the number and diameter of the myelinated fibers was higher in the treated group.
	Kokkalis et al. (2009)	Rats	Distal to end-to-side transfer	25	The ability of the acetyl-L-carnitine to enhance nerve regeneration after end-to-side neurorrhaphy in combination with various types of donor nerve injury distal to the coaptation site	Administration of acetyl-L-carnitine alone did not prove to be a stimulus, but in an injury model of the donor nerve (crush injury) proved to be a significantly more potent stimulus for regeneration.
	Wilson et al., 2010	Rats	Sciatic nerve	10	Adjuvant acetyl-L-carnitine treatment after transection and repair may improve both sensory and motor outcomes and merits further investigation.	Increases the number of regenerating nerve fibers but also morphologically improves the quality of regeneration and target organ weight and reinnervation signs.
Vitamin B group	Altun and Kurutas (2016)	Rats	Sciatic nerve	80	Tissue levels of vitamin B complex and vitamin B_12_ vary with progression of crush-induced peripheral nerve injury.	
	Kong et al. (2004)	Rats	Optic nerve	24	Intramuscular injections immediately after crush injury and then every 2 days demonstrated the potential for vitamin B_12_ as a neuroprotective agent after optic nerve crush injury.	Axons preservation was noted and more axons and retinal ganglion cells remained in the treated group.
	Kang et al. (2019)	rats	Sciatic nerve	9 (11)	Folic acid might improve peripheral nerve injury repair.	Promotes Schwann cell proliferation, migration, and secretion of nerve growth factor
Vitamin D	Chabas et al. (2013)	Rats	Peroneal nerve	36	Cholecalciferol induces a significant locomotor and electrophysiological recovery. Cholecalciferol increases number of preserved or newly formed axons in the proximal end mean axon diameter in the distal end neurite myelination in both distal and proximal ends	Acts on myelination *via* the activation of several myelin-associated genes involved in axogenesis and myelination.
Vitamin E	Tamaddonfard et al. (2014)	Rats	Sciatic nerve	60	Vitamin E (from Safranal) produced improving effects on crushed-injured sciatic nerve functions.	These effects may be mediated through antioxidant effects by reducing MDA level.
	Lu et al. (2011)	Mice	Spared Nerve Injury Model^a^	32	Neuropathic pain developed after peripheral nerve injury may be inhibited by combination of vitamin C and vitamin E.	Probably through the antioxidative and anti-inflammatory effects.
Magnesium	Pan et al. (2011)	Mice	Sciatic nerve	18	Improved neurological function recovery and enhanced nerve regeneration were found in mice with a sciatic nerve injury. Schwann cells may have been rescued from apoptosis by the suppression of inflammatory responses.	Mg supplementation improves: neurobehavioral, electrophysiological functions, enhanced regeneration marker, and reduced deposits of inflammatory cells as well as expression of inflammatory cytokines. Reduces Schwann cell apoptosis was in line with the significant expression of bcl-2, bcl-X (L) and down-regulated expression of active caspase-3 and cytochrome C.
Supplementation						
Epigallocatechin-3-gallate (in Green tea)	Renno et al. (2006)	Rats	Sciatic nerve	30	Epigallocatechin-3-gallate therapy has neuroprotective effects against trauma induced degeneration.	The mechanism of action of GT in reducing locomotion deficits and hyperalgesia that are often associated with peripheral nerve injury, is yet to be discovered.
	Kian et al. (2018)	Rats	Sciatic nerve	56		
Evening primrose oil	Ramli et al. (2017)	Rats	Sciatic nerve	72	Evening primrose oil supplementation improved peripheral nerve recovery in rats, by preserving: the shape of the axons the thickness of the myelin sheath the diameter of the axons	Acts *via* substrate for production of vasodilator prostanoids such as prostacyclin to improve nerve perfusion.
Sesame oil	Hsu et al. (2016)	Mice	Sciatic nerve	30	Sesame oil improved electrophysiological and functional assessments in mice with sciatic nerve crush injury, having beneficial effects on sciatic nerve regeneration and functional recovery.	Sesame oil significantly decreased lipid peroxidation and increased Nrf2 and GAP43 expression in sciatic nerve.
Melatonin	Rateb et al. 2017	Rats	Sciatic nerve	40	Melatonin had a significant role in improvement of the recovery of damaged sciatic nerves in rats. Especially when given at the dark period.	Through its antioxidant, antiapoptotic, anti-inflammatory effects and nerve growth factor stimulation.
	Pan et al. (2021)	Rats	Sciatic nerve	48	Proliferation and migration abilities of schwann cells in the melatonin group were significantly higher than those of Schwann cells in the control group	Bioinformatics analysis showed that Shh may be the key gene for the promotion of peripheral nerve regeneration by melatonin
Creatine	Helvacioglu et al. (2018)	Rats	Sciatic nerve	15	In rats, supplementation with creatine had a positive effect on the regeneration of injured sciatic nerve.	Probably diminishes the harmful effects of peripheral nerve crush injury.

^a^ The Spared Nerve Injury model involves ligation of two of the three branches of the sciatic nerve (the tibial nerve and the common peroneal nerve), while the sural nerve is left intact.

### 3.1. Diet

#### 3.1.1. Balanced diet

The lifelong alleviated diet was previously referred to as the attenuation of lipid peroxidation, inflammation, and immune cell infiltration, thus acting neuroprotective, preventing age-related damage to the nerves (9, 10).

The dietary components may have effects on PN alone or in combination. The synergistic effect may be enhanced with the consumption of nutrients and bioactive components together, also supported by the production of endogenous neurotrophic factors that increase environmental nerve repair (6).

#### 3.1.2. Energy value

Studies on the brain, spinal cord, and nerve regeneration have previously related low-calorie diet and hunger to improved regeneration (11). Limitation of energy intake in these patients is however complicated, and advising the patients to limit their energy intake is at least controversial.

#### 3.1.3. Obesity

Obesity may negatively affect PN regeneration after injury, through the reduction of growth factors amounts or their effectiveness, resulting in the delay of the regeneration and recovery.

The study of morphological features revealed significant differences in nerve structure and regeneration caused by the negative effects on axon number, myelin thickness, nerve area, the amplitude of compound action potential, and reduction in the number of growth factors in the sciatic nerve-injured rats due to fat-diet induced obesity (12).

#### 3.1.4. Alcohol

Neuropathy due to alcohol abuse is a known entity (13). Painful peripheral neuropathy resulting from the excessive and chronic use of alcohol occurs due to an unknown pathophysiological mechanism (14). Apart from malnutrition and nutrient deficiency occurring due to malabsorption, the direct neurotoxic effect was shown to be an independent factor in the development of the disease (15).

The same factors influence the regeneration of the nerve after injury. The study on rat models has demonstrated the negative impact of alcohol in rats with transected nerves (16).

These effects were previously evaluated in the Danish study by Behse and Buchtal who have compared the groups with alcoholic neuropathy and malnutrition neuropathy (17). In this study, Danish beer was the predominant alcoholic beverage, fortified with thiamine and Vitamin B6 at that time, and resulted in an absence of malnutrition, further development of symptoms and even led to weight gain. Alcoholic neuropathy group symptoms were related to pain, while the malnutrition group experienced progressive weakness, casting a decent shadow on the direct neurotoxic effect of alcohol *per se* (18).

### 3.2. Macronutrients

#### 3.2.1. Carbohydrates and lipids

The type and quantity of lipids may influence the regeneration of the PN by several mechanisms including pro-regenerative and neuroprotective, as well as pro-inflammatory and neurodegenerative effects when intake exceeds reasonable amounts.

A ketogenic diet had been attributed to a neuroprotective effect on PN, although more clinical trials are needed to prove the effects (19).

There is no specific recommendation, but the lipid content type and appropriate *n* − 6/*n* − 3 ratio seem to make a significant influence on nerve regeneration. Omega-3 and omega-9 polyunsaturated fatty acid's positive effects were demonstrated.

##### 3.2.1.1. Omega-3 fatty acids

Experimental studies support the use of polyunsaturated fatty acids as a promising pharmacological approach in PN injuries.

Polyunsaturated fatty acids, such as eicosapentaenoic and docosahexaenoic acids, mediate neuroprotective and pro-regenerative effects, while also inhibiting neuroinflammation and oxidative stress. The exact mechanism is not known, and it is considered to be a combination of targets, from ion channels to nuclear receptors (20).

In mice, oral administration of combined eicosapentaenoic and docosahexaenoic acids had regenerative and possibly protective properties after PN injury (21).

##### 3.2.1.2. Omega-9 fatty acids

CIS-monounsaturated omega-9 fatty acid—oleic acid, administered in combination with albumin or as 2-hydroxyoleic acid, promotes antinociception and anxiolytic effects following both central and PN injury. Although these results are generally positive, the impact on regeneration is questionable. Motor function improvement and spasticity reduction were observed in patients with spinal cord injury, implicating the possibility of a positive effect on PN (22).

##### 3.2.1.3. Alpha-lipoic acid

In rats with sciatic nerve injury, alpha-lipoic acid has a protective effect through the improvement of regeneration of the injured nerve by its antiapoptotic and anti-inflammatory effects (23), while the implantation of composite nanofiber sheets incorporating alpha-lipoic acid and atorvastatin contributed to the recovery of the motor and sensory function and nerve regeneration (24). As a treatment for neuropathic pain caused by PN injury potentially is considered alpha-lipoic acid, which requires further verification (25).

#### 3.2.2. Proteins and amino acids

The consumption of protein resources with high biological quality, especially including essential amino acids must, be maintained at a certain level to meet organism requirements (6).

The specific role in augmentation is reflected by the effects of natto (extracts of fermented soybeans) on the improvement of motor function and in electrophysiological results, through a complex nerve preservation mechanism (26).

##### 3.2.2.1. Leucin

The study of Singer and Mehler, also tried to explain the increased leucine uptake in fasted animals. The two options included (1) deficit of amino acids produced by fasting, inducing or derepressing enzymes, which transport leucine, and (2) amino acid metabolism depression because of the deficit of glucose (resulting in a deficit of metabolic products of amino acids metabolism which could accelerate uptake) (27).

##### 3.2.2.2. Carnosine

The gastrocnemius muscle mass reduction from a sciatic nerve crush injury may be improved near to its normal value with carnosine supplementation. The beneficial effects may be associated with the acceleration of functional recovery and the improvement of histological and ultrastructural alterations through the mechanisms of suppression of lipid peroxidation, provoking of antioxidant enzyme activity and amelioration of cytokine production (28).

#### 3.2.3. Acetyl-L-carnitine

The modified amino acid is one of the most researched nutrients in peripheral nerve injuries and their surgical treatment. Several studies carried out in animals were published on the various applications of acetyl-L-carnitine for nerve preservation (29), regeneration (30), and augmentation of surgical treatment by both topical (31) and systemic administration (32), even in the delayed fashion (33).

The results of all these studies demonstrated beneficial effects on regeneration; however, the neuroprotective effects were not as pronounced, especially when the acetyl-L-carnitine was given in a delayed fashion (7 days after injury). A recent study evaluated individual and combined effects of erythropoietin and acetyl-L-carnitine; however, regardless of the positive impact from both, combined improved efficacy was not found (34).

The only human study related to nutritional therapy failed to stress the importance of diet nutrition and supplementation in nerve regeneration, although in a chronic entrapment, not the injury. In this double-blinded, randomized, placebo-controlled study, which included adult patients with severe carpal tunnel syndrome acetyl-L-carnitine did not improve nerve regeneration (35).

### 3.3. Micronutrients

#### 3.3.1. Vitamins B6 and B12

The beneficial effects of group B6 and B12 vitamins in peripheral neuropathies are well known. The possibility and impact of the improvement of nerve regeneration after injury are not sufficiently clarified. It was previously shown that the levels of the vitamin B complex are significantly lower immediately after the injury, with progression through time Supplementation of vitamin B complex in the acute period of PN injury deserves to be considered as an option, which may be useful for the acceleration of nerve regeneration (36). However, the mechanism of action in injured nerve regeneration is different, due to the different types of nerve lesions. Namely, the immediate damage, and the role of supplements is induction of regeneration, not protection (9).

Vitamin B12 (methylcobalamin) has an analgesic effect which may be explained by improving nerve conduction velocity and regeneration of injured nerve. Also, in neuropathic pain states, methylcobalamin inhibited the ectopic spontaneous discharges from peripheral sensory neurons (37).

The underlying pathophysiological mechanism is probably neuronal protection by promotion of regeneration of injured nerves (38) while antagonizing glutamate-induced neurotoxicity (37).

#### 3.3.2. Folic acid

Supplementation with folic acid improved the organoleptic features of spinal axons in *in vivo* grafted PN segments of adult Sprague-Dawley rats. The same positive effects were noted in spinal cord contusion injuries, emphasizing that the folic acid supplementation should not be limited only to the embryonic period and prevention of neural-tube defects, but also to augmentation of functional recovery in surgically treated PN injuries (39).

In rats, folic acid might improve PN injury repair. Namely, Schwann cells’ proliferation and migration, as well as nerve growth factors’ secretion were promoted by folic acid (40).

#### 3.3.3. Vitamin D

Vitamin D is known for its potential in immune response regulation in various diseases. In PN injury, the positive effect on myelination was demonstrated in rat models. This specific effect is intended to be used solely, although the use with the surgical repair might lead to a better functional recovery.

Chabas et al. on animal models have proven the potential of ergocalciferol (Vitamin D2) and cholecalciferol (Vitamin D3) in the augmentation of the spinal cord and PN regeneration. An analysis of the gene, which regulates Vitamin D3 in the ganglia of the back roots and Schwan's cells, was also performed.

Cholecalciferol is more effective than ergocalciferol and, when administered at a high dose (500 IJ/kg/day), cholecalciferol induces significant locomotor and electrophysiological recovery by increasing the number of preserved and newly formed axons at the proximal end, the mean diameter of the axon at the distal end, and induction of myelination at both distal and proximal ends.

A modified expression of several genes involved in axonogenesis and myelination was also found, after 24 h of vitamin D3 introduction, which leads to the conclusion that Vitamin D acts on myelinization by activating several associated genes (41).

#### 3.3.4. Vitamin E

One study, analyzing the impact of vitamin E, found some improving effects on motor impairment, pain hypersensitivity, Wallerian degeneration, and muscular atrophy induced by a sciatic nerve crush injury. The effect is probably due to the inhibition of the oxidative stress pathway by reducing the malondialdehyde level.

Due to the high contents of Vitamin E, safranal, a major component of saffron, is recommended as a dietary supplement in patients with nerve injury (42).

It was also previously demonstrated that neuropathic pain developed after PN injury may be inhibited by the combination of vitamin C and vitamin E, probably through the antioxidative and anti-inflammatory effects (43).

#### 3.3.5. Magnesium (Mg)

Magnesium supplements significantly improve functional recovery in various neurological disorders, especially in cerebrovascular disease through the decrease in systemic vascular resistance and improvement of cardiac function (44). Not as much data are available on PN injuries related effects, and even those studies involve filaments and wires, rather than supplementation (45, 46). In a study of the injury of the sciatic nerve in an animal model, the diet with high magnesium content significantly increased plasma and plasma magnesium concentrations. In addition, magnesium supplements improved neurobehavioral and electrophysiological functions, improved regeneration markers, and reduced inflammatory cell deposits, as well as the expression of inflammatory cytokines. Schwan cell cellular apoptosis was also reduced in accordance with significant expression of Bcl-2, Bcl-KSL and decreased expression of active Caspase-3 and cytochrome C. It was concluded that magnesium positively influences neurological regeneration and improves neural regeneration, while also preserving Schwan's cells of apoptosis by suppressing inflammatory response (47).

### 3.4. Supplementation

#### 3.4.1. Green tea

The intake of green tea may assist nerve recovery after traumatic injuries. Although more studies should explore the cellular and molecular mechanisms, which mediate such effect, initial studies with a green tea polyphenol epigallocatechin gallate led to some promising conclusions (48).

##### 3.4.1.1. Epigallocatechin gallate

Prior to the study on PN, a possible therapeutic effect in spinal cord injury was noticed through the improvement in the flat beam test. Furthermore, at an early stage of spinal cord injury, inflammatory cytokines were modulated and axonal sprouting was higher (49).

The study on sciatic nerve transection injury revealed biochemical, histopathological, and immunohistochemical evidence that epigallocatechin gallate therapy may have neuroprotective effects against injury-induced degeneration (50).

#### 3.4.2. Sesame oil

Polyunsaturated (omega-6), and monounsaturated (omega-9) fatty acids account for more than 80% of the total fat contents of sesame oil. Together with the natural antioxidant sesamol and vitamin E, the content assures a neuroprotective effect.

In a study by Hsu et al. sesame oil improved electrophysiological and functional assessments in mice with sciatic nerve crush. The beneficial effects are based on significantly decreased lipid peroxidation and increased Nrf2 and GAP43 expression in the sciatic nerve (51).

#### 3.4.3. Evening primrose oil

Supplementation with evening primrose oil might be significant in the therapy of PN injury. It was shown that evening primrose oil supplementation improved PN recovery in rats (52).

#### 3.4.4. Melatonin

Melatonin may improve the proliferation and migration of Schwann cells *via* the Sonic Hedgehog signaling pathway after PN injury and in that way promote PN regeneration (53). Melatonin had a significant role in the improvement of the recovery of damaged sciatic nerves in rats, through its antioxidant, antiapoptotic, anti-inflammatory effects, and nerve growth factor stimulation. Giving melatonin during the dark period had better results than giving melatonin during the light period (54).

#### 3.4.5. Creatine

In rats, supplementation with creatine had a positive effect on the regeneration of injured sciatic nerve, which was also verified by electronic microscopy (55).

### 3.5. Implications and limitations

Reviewed studies comprised different animal models and different options involving the dietary and nutritional interventions as well as the supplements used for the improvement of nerve preservation and regeneration after injury, as well as for the outcome augmentation following surgical repair.

Since the whole review is based on animal studies, we could not derive clear conclusions or recommendations for surgical treatment augmentation in the human population, but focused to give an insight into every possible dietary, nutritional or supplement-related influence.

Possible mechanisms of action are shown in Table 1 in detail and give a significant contribution to a deeper understanding of the general aspects of diet, nutrition, and supplementation effects on the peripheral nervous system.

**Figure 1:**
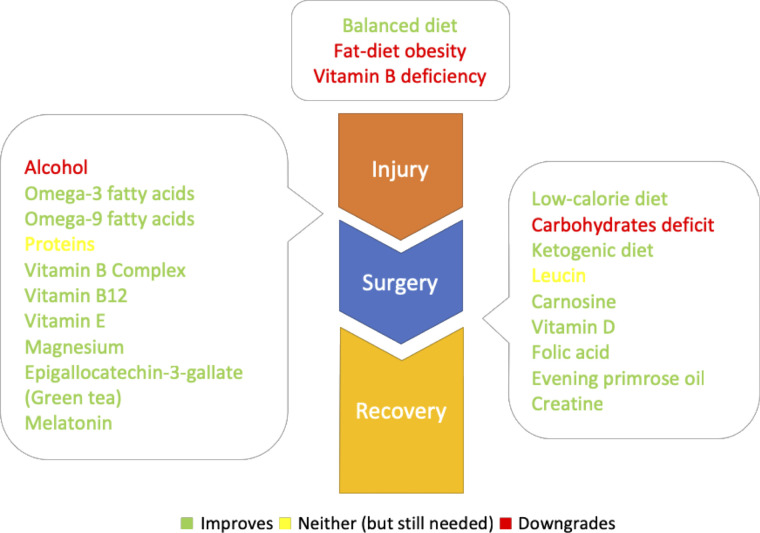
Diet, nutrients and supplements with their respective target points: up: predisposing factors; left: predominately neuroprotective (could be taken from the injury onset); right: predominately neuroregenerative (could be taken after the surgery).

To provide some initial waypoint, based on our review and the presumed mechanism of action, the specific target points for the introduction or activity of the individual nutrients and supplements were marked in Figure 1.

## 4. Conclusions

Standardized diet, nutrition, and supplementation protocols may be of the greatest importance for better nerve regeneration and functional recovery, as a part of the multidisciplinary approach to achieve the best possible results in patients with PN injuries in the future.

The augmentation of functional recovery, as a part of the multidisciplinary approach, is far less controversial than the treatment of PN injuries based entirely on conservative options. Further human studies should focus on the augmentation of functional recovery, in addition to the surgical treatment of PN injuries, to clarify the definite underlying mechanisms and give clear recommendations and guidelines for better outcomes.

## Data Availability

The original contributions presented in the study are included in the article/Supplementary Material, further inquiries can be directed to the corresponding author.
